# Silencing of *TaCKX1* Mediates Expression of Other *TaCKX* Genes to Increase Yield Parameters in Wheat

**DOI:** 10.3390/ijms21134809

**Published:** 2020-07-07

**Authors:** Bartosz Jabłoński, Hanna Ogonowska, Karolina Szala, Andrzej Bajguz, Wacław Orczyk, Anna Nadolska-Orczyk

**Affiliations:** 1 Department of Functional Genomics, Plant Breeding and Acclimatization Institute—National Research Institute, Radzikow, 05-870 Blonie, Poland; b.jablonski@ihar.edu.pl (B.J.); h.ogonowska@ihar.edu.pl (H.O.); k.szala@ihar.edu.pl (K.S.); 2 Laboratory of Plant Biochemistry, Faculty of Biology, University of Bialystok, Ciolkowskiego 1J, 15-245 Bialystok, Poland; abajguz@uwb.edu.pl; 3 Department of Genetic Engineering, Plant Breeding and Acclimatization Institute—National Research Institute, Radzikow, 05-870 Blonie, Poland; w.orczyk@ihar.edu.pl

**Keywords:** wheat, cereals, *TaCKX1*, *TaCKX* expression, grain yield, cytokinins, phytohormones, gene silencing, RNAi, wheat spikes

## Abstract

*TaCKX*, *Triticumaestivum* (cytokinin oxidase/dehydrogenase) family genes influence the development of wheat plants by the specific regulation of cytokinin content in different organs. However, their detailed role is not known. The *TaCKX1*, highly and specifically expressed in developing spikes and in seedling roots, was silenced by RNAi-mediated gene silencing via *Agrobacterium tumefaciens* and the effect of silencing was investigated in 7 DAP (days after pollination) spikes of T_1_ and T_2_ generations. Various levels of *TaCKX1* silencing in both generations influence different models of co-expression with other *TaCKX* genes and parameters of yield-related traits. Only a high level of silencing in T_2_ resulted in strong down-regulation of *TaCKX11 (3)*, up-regulation of *TaCKX2.1*, *2.2*, *5,* and *9* (*10*), and a high yielding phenotype. This phenotype is characterized by a higher spike number, grain number, and grain yield, but lower thousand grain weight (TGW). The content of most of cytokinin forms in 7 DAP spikes of silenced T_2_ lines increased from 23% to 76% compared to the non-silenced control. The CKs cross talk with other phytohormones. Each of the tested yield-related traits is regulated by various up- or down-regulated *TaCKX* genes and phytohormones. The coordinated effect of *TaCKX1* silencing on the expression of other *TaCKX* genes, phytohormone levels in 7 DAP spikes, and yield-related traits in silenced T_2_ lines is presented.

## 1. Introduction

Wheat (*Triticum aestivum* L.) is the third most economically important crop in the world after corn and rice, and probably the most important in moderate climates. It provides approximately 20% of human calories and protein [1]. The large genome of this high-yielding species, composed of three (AABBDD) genomes, has been very challenging for improving traits [2]. However, it might be a great reservoir to sustain a further increase of grain productivity [3]. The continuous increase of wheat production is necessary to feed the rapidly growing world population [4]. Biotechnological tools implemented in the process of increasing wheat productivity are expected to be beneficial. 

Cytokinins (CKs) are important regulators of plant growth and development, influencing many agriculturally important processes [5]. This regulation might occur at the posttranscriptional and/or posttranslational level [6,7], or by the modulation of context-dependent chromatin accessibility [8]. CKs modulate the expression of other genes involved in the control of various processes including meristem activity, hormonal cross talk, nutrient acquisition, and various stress responses [9]. There is growing evidence on their key role in seed yield regulation [10]. In cereals and grasses, an increased content of CKs has been reported to positively affect sink potential in developing grains [11] and maintain leaf chlorophyll status during plant senescence [12] and grain filling [13]. 

The majority of naturally occurring CKs in plants belong to isoprenoid cytokinins grouping *N*^6^-(12-isopentenyl) adenine (iP), *trans*-zeatin (tZ), *cis*-zeatin (cZ), and dihydrozeatin (DZ) derived from tRNA degradation or from isopentenylation of free adenine nucleosides catalysed by isopentenyltransferase (IPT) or tRNA-IPT. The second, smaller group comprise N6-aromatic CKs, represented by benzyladenine (BA) [14]. To better characterize their physiological role, CKs are classified into such -base active forms as tZ, cZ, and iP, translocation forms (nucleosides) as tZ-ribosides (tZR), which exhibit a low level of activity, and sugar conjugates (*O*-glucosides), which are storage and inactivated forms [14,15].

CKs function as local or long-distance regulatory signals, but the mechanisms of their precise spatial and temporal control are still largely unknown [16]. They are produced in roots as well as in various sites of the aerial part of plants [17]. The level of CKs in respective cells and tissues is dependent on many processes, including biosynthesis, metabolism, activation, transport, and signal transduction. Active CKs can be metabolized via oxidation by cytokinin oxidase/dehydrogenase (CKX) or by activity of glycosyltransferases. Many reports have demonstrated that the irreversible degradation step by the CKX enzyme plays an important role in the regulation of cytokinin level in some cereals, namely maize [18], rice [19], barley [20,21], and wheat [22].

The *CKX* gene families in plants show different numbers of genes and various expression patterns, which are tissue- and organ-specific, suggesting gene-specific functions. The specificity of expression of 11 *TaCKX* in developing wheat plants were assigned to four groups: highly specific to leaves, specific to developing spikes and inflorescences, highly specific to roots and expressed through all the organs tested [23]. The *TaCKX* genes co-operated inside and among organs. Their role in plant productivity has been described in many plants including model plants and some cereals. Knock-out mutation or silencing by RNAi of *OsCKX2* in rice significantly increased grain number [19]. The same effect of elevated grain number, spike number, and yield was reported for RNAi-silenced *HvCKX1* in barley [20,21,24] and repeated for the same gene under field conditions [25]. Moreover, significantly increased grain number per spike was found as the effect of the *TaCKX2.4* gene silencing by RNAi [26]. Knock-out mutation of *HvCKX1* by CRISPR/Cas9 editing had a limited effect on yield productivity, however significantly decreased CKX enzyme activity in young spikes and 10-day old roots corresponded to greater root length, numbers of root hairs and increased surface area [27]. In contrast, roots of knock-out mutants of ckx3 were smaller. 

The role of other *TaCKX* genes in wheat was analysed based on natural *TaCKX* variation. Haplotype variants of *TaCKX6a02* and *TaCKX6-D1* were related to higher filling rate and grain size [28,29]. Quantitative trait locus (QTL) found in recombinant inbred lines containing a higher copy number of *TaCKX4* was associated with higher chlorophyll content and grain size [30]. 

To arrange the numbering of *TaCKX* family genes, a new annotation for the first two was suggested by Ogonowska et al. (2019) based on the Ensembl Plants database [31] and phylogenetic analysis. *TaCKX6a02* was annotated as *TaCKX2.1*, *TaCKX6-D1* (JQ797673) was annotated as *TaCKX2.2* and *TaCKX2.4* was annotated as *TaCKX2.2*. Annotations for these genes were maintained in the recently published review on the *TaCKX* [22], however tested in this research *TaCKX10* was renamed as *TaCKX9* and *TaCKX3* was renamed as *TaCKX11*. Newly revised by Chen et al. [22], naming is applied and former names are given in brackets.

Due to the size and complexity of the wheat genomes, the knowledge about the role of *TaCKX* genes, containing homologues from three genomes, is more difficult to obtain, because of the limited number of natural mutants. Most homoeologous genes are expected to have overlapping functions [32], therefore the effect of gene mutations might be masked by the other genomes. One solution to silence all of them is to apply RNAi-mediated gene silencing, which allows silencing of all the homologues. Moreover, this tool made it possible to obtain a number of lines with different levels of silencing, which in the case of genes coding proteins of key importance for life gave a possibility to regenerate plants for analysis [33]. The introduction of a silencing cassette by stable transformation results in a stable, and inherited to T_4_, effect of silencing [21,34]. The applicability of *Agrobacterium*-mediated transformation compared to a biolistic one for gene silencing of the developmentally regulated gene *HvCKX10* (*2*) was proved to be reliable [24]. 

We present the first report on the role of *TaCKX1* in the co-regulation of expression of other *TaCKX* genes, phytohormone content, and their joint participation in the regulation of yield-related traits in wheat. Various levels of gene silencing in T_1_ and T_2_ have been related to different patterns of other *TaCKX* expression, strongly influencing yield-related traits. Models of regulation of phytohormone levels and phenotypic traits in non-silenced and highly silenced T_2_ plants by the coordinated expression of *TaCKX* genes are proposed.

## 2. Results

### 2.1. Expression Levels of Silenced TaCKX1 in Segregating T_1_ and T_2_ Plants

**Figure 1:**
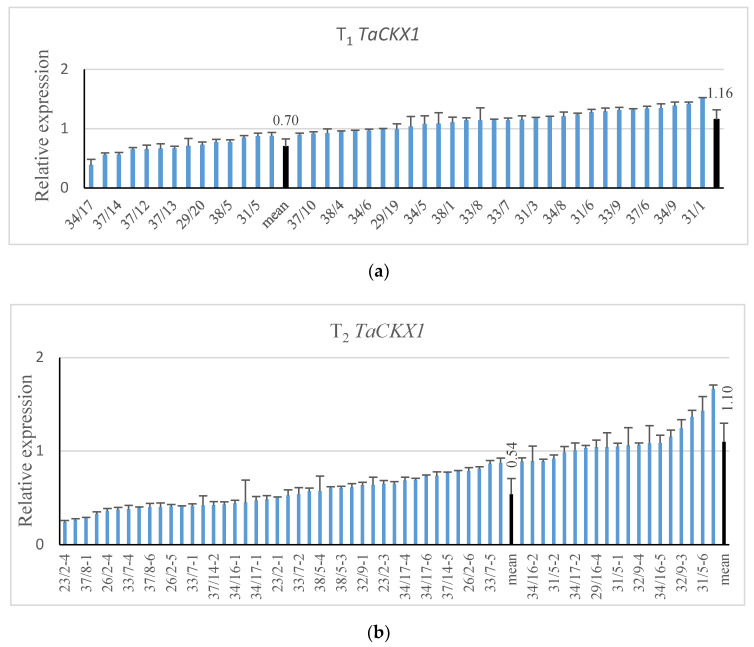
Relative expression level of silenced *TaCKX1* in segregating T_1_ (**a**) and T_2_ (**b**) plants. The level of expression is related to the control set as 1.00.

Expression levels of *TaCKX1* were measured in 44 segregating T_1_ plants from 8 T_0_ PCR+ lines. In 14 T_1_ plants relative expression (related to the control = 1.00) ranged from 0.39 to 0.88 with the mean of 0.67 (±0.14). In 30 T_1_ plants, relative expression ranged from 0.90 to 1.52 with the mean of 1.16 (±0.18) (Figure 1). The proportion of silenced to non-silenced plants changed in the T_2_ generation. There were 42 silenced from 0.24 to 0.88 plants with the mean of 0.54 (±0.14) and 20 non-silenced plants. Eight of them, with low relative expression ranging from 0.24 to 0.40 (mean 0.33 ±0.14) and representing different T_1_ lines, were selected for further analysis.

### 2.2. Co-Expression of Silenced TaCKX1 with Other TaCKX Genes in T_1_ and T_2_ and CKX Enzyme Activity

**Figure 2:**
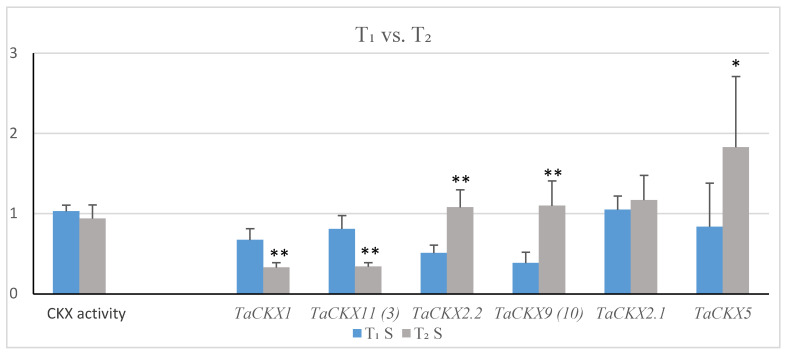
Comparison of means of relative CKX enzyme activity and selected gene expression levels in T_1_ (bars) and T_2_ (line) generation of silenced lines. *—significant at *p* < 0.05; **—significant at *p* < 0.01.

Mean relative expression of *TaCKX1* in the selected 8 lines was 0.67 in T_1_ and was decreased to 0.33 in T_2_ (Figure 2). Similarly, in the case of *TaCKX11* (*3*) related gene expression was 0.81 in T_1_ and was decreased to 0.34 in T_2_. Relative expression levels of *TaCKX2.2* and *TaCKX9* (*10*) were decreased in T_1_ to 0.51 and 0.39 and increased in T_2_ slightly above the control level, to 1.08 and to 1.10 respectively. Mean relative values for *TaCKX2.1* were similar to control in T_1_ (1.05) and slightly increased in T_2_ (1.17). Relative expression of *TaCKX5*, which was in T_1_ below the control level (0.84), was significantly increased to 1.82 in T_2_. The relative values of CKX enzyme activity in both generations were around the control, 1.00.

The effect of *TaCKX1* silencing on the levels of expression of selected *TaCKX* genes is presented by the expression ratio indicator (Table 1), which is a quotient of the mean relative value in silent per mean relative value in non-silent, control plants. In the case of *TaCKX1* and *TaCKX11* (*3*), the ratio indicator, significantly decreased in T_1_, was strongly decreased in T_2_. The value of the ratio indicator for *TaCKX2.2* was not changed in T_1_ compared to the control and was only slightly decreased in T_2_. The expression ratio indicator of *TaCKX9 (10)*, strongly decreased to 0.59 in T_1_, rose above the control level (1.15) in T_2_. Already high in T_1_, the expression ratio indicator for *TaCKX2.1* (1.22) increased to 1.32 in T_2_. The phenotype ratio indicator for CKX enzyme activity was 1.01 in T_1_ and 0.99 in T_2_. 

In T_1_ segregating plants, CKX enzyme activity significantly correlated with spike length (0.51; n = 16) and grain weight (0.50; n = 16), but in T_2_ these correlations were not significant.

### 2.3. Influence of TaCKX1 Silencing on Phenotypic Traits and Chlorophyll Content in Flag Leaves of T_1_ and T_2_ Plants 

The values of phenotypic traits in T_1_ plants with slightly decreased relative expression of *TaCKX1* (0.67 ± 0.14) compared to control plants (1.00) were on the same level in the case of plant height and lower for number of spikes, spike length, grain number, and grain yield (Supplementary Table S2). Higher values were obtained for TGW. Data for chlorophyll content measured by SPAD in the flag leaves of first spikes and the next spikes were similar. All these differences were not significant. Opposite results were obtained for some traits in T_2_ plants with highly silent *TaCKX1* (0.33 ± 0.06) compared to the control (1.00) (Supplementary Table S3). Silent T_2_ plants were substantially smaller, had a higher number of spikes, number of grains, grain yield, seedling root weight, and lower SPAD values for the flag leaves of first spikes. TGW and spike length were significantly lower than in control plants.

**Figure 3:**
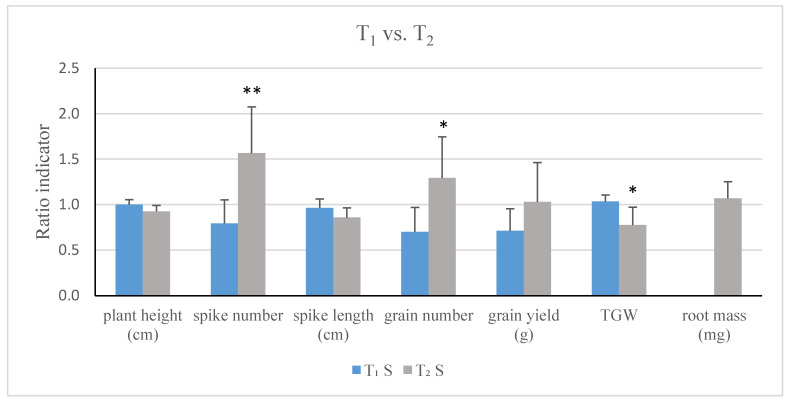
Comparison of phenotypic effect of silencing of *TaCKX1* in T_1_ and T_2_ generations based on ratio indicators. *—significant at *p* < 0.05; **—significant at *p* < 0.01.

These differences between the slightly silenced T_1_ and highly silent T_2_ generation are expressed by comparison of ratio indicators of phenotypic traits in both generations (Figure 3). There were no changes in plant height, TGW or spike length in T_1_ plants compared to the control. However, these values were respectively 7%, 10%, and 25% lower in T_2_ plants. Opposite phenotype ratio indicators for number of spikes per plant and number of grains per plant were about 21% and 30% lower in T_1_ and 57% and 29% higher in T_2_. These differences for spike number, grain number, and TGW were significant.

The levels of expression of *TaCKX1* in 7 DAP spikes of all T_1_ significantly correlated with number of grains, grain weight, spike length and spike number (0.47, 0.39, 0.42 and 0.33 respectively; n = 42) and grain weight correlated with enzyme activity (0.33; n = 42). The *TaCKX9 (10)* expression level significantly correlated with grain number (0.51; n = 16). 

Correlation coefficients among the expression of all tested *TaCKX* genes and enzyme activity, and phenotypic traits in non-silent and highly silent T_2_ are included in Supplementary Table S4A,B. All these correlations are graphically presented in Figures and described in Section 2.6.

### 2.4. Phytohormone Content in 7 DAP Spikes of T_2_

**Figure 4:**
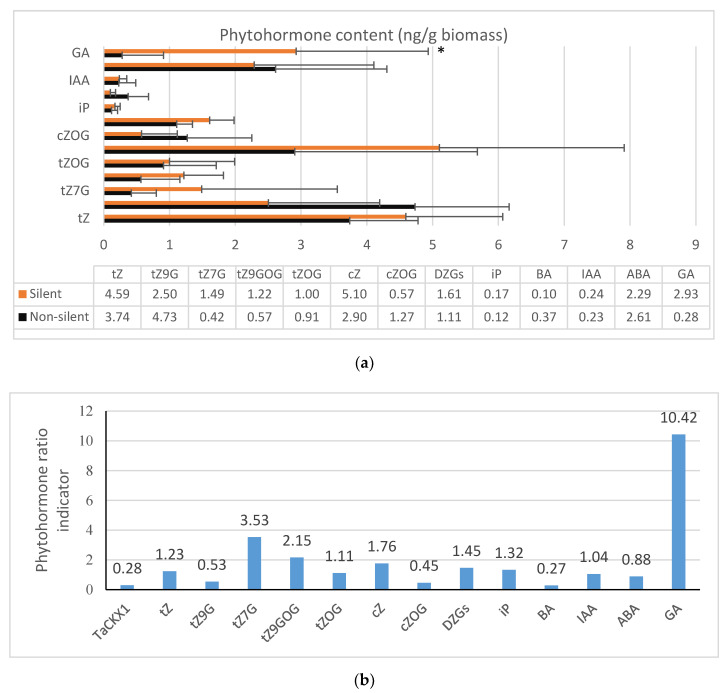
Phytohormone content (ng/g biomass) measured in the group of control and silent T_2_ plants (**a**). Phytohormone ratio indicators (mean value in silent per mean value in not silent, control plants) in silent T_2_ plants (**b**). *—significant at *p* < 0.05. Small amounts (≤1.00 ng/g biomass): tZR, tZOGR, cZOGR, DZOG, DZ7G, DZ9G, DZOGR, iP, iP7G, BA, IAA. Trace amounts (≤0.05 ng/g biomass) or not detected: cZ9G, cZR, DZ, DZR, iPR, IBA, IPA, NAA, PAA.

tZGs, which were mainly composed of tZ9G, tZ7G, tZOG and tZ9GOG, were the most abundant cytokinin group in 7 DAP spikes (Figure 4a). Their mean content in control plants was 6.97 ng/g biomass and in silent T_2_ was 6.24 ng/g biomass respectively. The second most abundant was tZ with the level of 3.74 ng/g biomass in the control and 4.59 ng/g biomass in silent T_2_. The content of cZ was slightly lower to tZ (2.90 ng/g biomass) in control but higher (5.10 ng/g biomass) in silent plants. cZOG was more abundant in the control than the groups of silent plants, and the content was 1.27 and 0.57 ng/g biomass respectively. The concentration of DZGs (sum of DZ7G, DZOG, DZ9G and DZOGR) was higher in silent (1.61 ng/g biomass) than in control plants (1.11 ng/g biomass). Low concentrations (below 0.5 ng/g biomass) were measured for iP and BA. The concentration of IAA was also low and on a comparable level in control and in silent plants (0.23 and 0.24 ng/g biomass respectively). In the case of ABA, the concentration in the control was slightly decreased in silent plants (2.61 and 2.29 ng/g biomass respectively). The concentration of GA was increased from 0.28 ng/g biomass in the control to 2.93 ng/g biomass in silent plants, which was more than a 10-fold increase.

Most of the phytohormone ratio indicators in the group of six silent T_2_ plants (Figure 4b) were much higher than in control plants. There were the following cytokinins: tZ (1.23), tZ7G (3.53), tZ9GOG (2.15), tZOG (1.11), cZ (1.76), sum of DZGs (1.45) and iP (1.32). The ratio indicators for some of them were significantly lower, as in the case of BA (0.27), cZOG (0.45) and tZ9G (0.53). Similar values were observed for IAA (1.04), and slightly lower for ABA (0.88), but much higher for GA (10.42).

### 2.5. Coordinated Effect of TaCKX1 Silencing on Expression of Other TaCKX Genes and Phytohormone Level in 7 DAP Spikes as Well as Phenotype in T_2_

**Figure 5:**
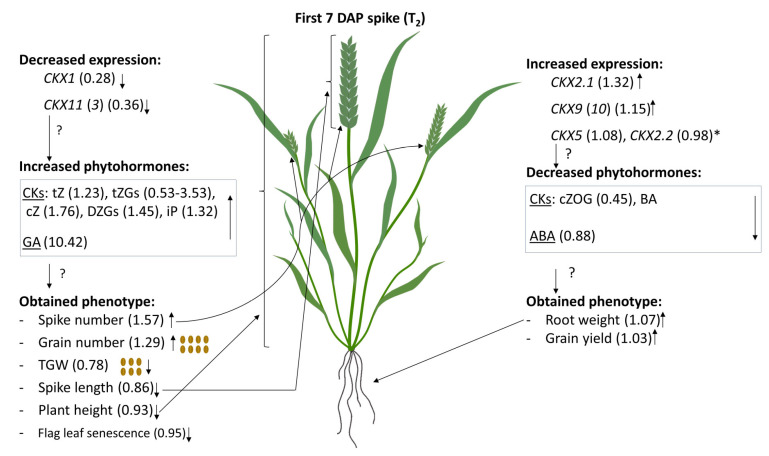
Graphic presentation of coordinated effect of *TaCKX1* silencing on expression of other *TaCKX* genes, phytohormone levels as well as phenotype in 7 DAP spikes of T_2_ plants based on ratio indicators. *—significantly increased comparing to T_1_; ?—expected changes.

A graphic presentation of the coordinated effect of *TaCKX1* silencing on expression of other *TaCKX* genes and phytohormone levels in 7 DAP spikes as well as the phenotype of T_2_ plants is presented in Figure 5. The significant decrease of expression of *TaCKX1* was coordinated with the significant decrease of *TaCKX11* (*3*), which presumably resulted in a significant increase of most CKs: tZ, tZGs, cZ, DZGs, iP, as well as GA. The increased phytohormone level in the first 7 DAP spikes positively influenced traits such as spike number and grain number, reaching the ratio indicators 1.57 and 1.29, respectively, and negatively influenced TGW (0.78), spike length (0.86), plant height (0.93), and flag leaf senescence (0.95). Opposing data were obtained for *TaCKX2.1* and *TaCKX9* (*10*), which showed increased expression in silenced 7 DAP spikes (1.32 and 1.15 respectively). This might have influenced the decreased ratio indicators for phytohormones—cZOG (0.45), BA (0.27), and ABA content (0.88), and slightly increased ratio indicators for yield-related traits: root weight and grain yield (1.07 and 1.03 respectively). Expression ratio indicators for *TaCKX5* and *TaCKX2.2* were both close to 1.00, but their expression significantly increased compared to T_1_ and positively correlated with the expression of *TaCKX2.1* and *TaCKX9* (*10*) respectively.

### 2.6. Models of Co-Regulation of Phytohormone Levels and Phenotype Traits by Coordinated Expression of TaCKX Genes in Non-Silenced and Silenced T_2_ Plants 

**Figure 6:**
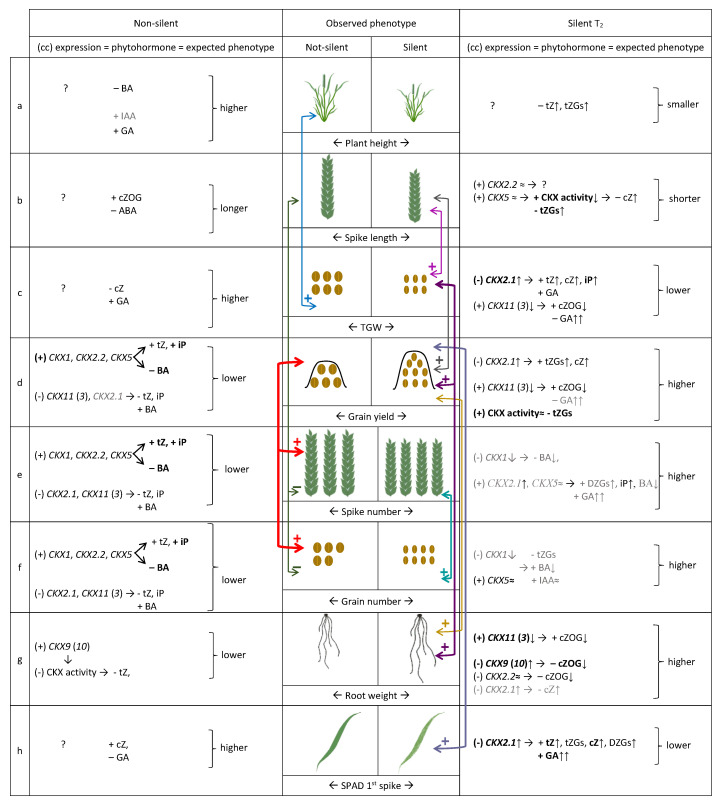
Models of regulation of phytohormone levels and phenotypic traits by coordinated expression of *TaCKX* genes based on correlation coefficients (cc) in non-silenced and silenced wheat plants (**a**–**h**). (cc)—correlation coefficient between expression and trait; (?) – lack of correlation with expression of any gene; bold—strong, significant correlations at *p* ≤ 0.05 (cc above 0.82); grey—cc from 0.5 to 0.6.

Two different models of co-regulation of *TaCKX* expression, phytohormone levels and phenotypic traits in non- silenced and silenced plants of the T_2_ generation are proposed (Figure 6a–h) based on correlation coefficients (Table S4A,B). 

Plant height (Figure 6a). There was no correlation between plant height and expression values of any *TaCKX* expressed in 7 DAP spikes of non-silent as well as silent plants. In the first group of plants this trait negatively correlated with BA and positively with IAA and GA content. By contrast, in silent plants the values of plant height were negatively correlated with growing concentration of tZ and tZGs, which resulted in a smaller plant phenotype.

Spike length (Figure 6b) in non-silent plants was positively correlated with BA, and negatively with cZ and ABA content. These correlations determined longer spikes and the trait negatively correlated with spike number and grain number. A strong positive correlation between CKX activity and spike length was noted in silent plants. The values of enzyme activity correlated positively with slightly increased *TaCKX5* expression, which negatively correlated with increasing content of cZ and tZGs. Spike length in silent plants was positively correlated with grain yield.

TGW (Figure 6c). There was no correlation of TGW with expression of any *TaCKX* expressed in 7 DAP spikes of non-silent plants. However, the trait was strongly negatively correlated with cZ content and positively with GA. The grains in this group of plants were larger and TGW higher. By contrast, in silent plants there was a strong negative correlation of the trait with growing expression of *TaCKX2.1*, which positively regulated tZ, cZ, iP, and GA content. Moreover, the values of expression of down-regulated *TaCKX11* (*3*) positively correlated with decreasing content of cZOG, negatively with highly growing GA and positively with the trait. Altogether it resulted in lower TGW compared to non-silenced plants. The trait in silent plants was strongly and positively correlated with grain yield (0.82) and root weight (0.77).

Grain yield (Figure 6d). Expression levels of *TaCKX1*, *TaCKX2.2* and *TaCKX5* in non-silent plants positively correlated with tZ and iP and negatively with BA content. However, expression of *TaCKX11* (*3*) and *TaCKX2.1* regulates the same CKs in opposite way. Altogether, it resulted in lower grain yield comparing to silenced plants, and the trait was strongly positively correlated with spike number (0.93) and grain number (0.99). The increasing expression of *TaCKX2.1* positively correlated with a growing content of tZGs and cZ and negatively with the trait in silent plants. Decreasing expression of *TaCKX11* (*3*), which was positively correlated with decreased cZOG content and negatively with GA content, positively correlated with the trait. A positive correlation was observed between CKX activity and grain yield in this group of plants, which was higher than in non-silent plants. Moreover, CKX activity negatively correlated with tZGs. The trait was strongly correlated with TGW (0.82) and root weight (0.66).

Spike number (Figure 6e) and grain number (Figure 6f) in non-silenced plants were positively regulated by *TaCKX1*, *TaCKX2.2* and *TaCKX5*, and their expression was positively correlated with tZ, iP and negatively with BA. On the other hand, expression levels of *TaCKX2.1* plus *TaCKX11* (*3*) were negatively correlated with the traits as well as with tZ, iP and positively with BA. Both groups of genes finally affected lower spike and grain numbers in non-silent plants in comparison to silent plants and were strongly and positively correlated with each other (0.91) and grain yield (0.93 and 0.99 respectively). In silent plants decreasing expression of *TaCKX1* is negatively correlated with both spike and grain number and the gene negatively regulates decreasing BA content. In the case of grain number, the main player positively correlated with the trait is *TaCKX5*, increased expression of which was correlated with slightly higher IAA content, which resulted in higher grain number. Spike number is also positively regulated by *TaCKX5* co-expressed with *TaCKX2.1*, and both genes were positively correlated with growing CKs, DZGs and iP as well as GA, determining higher spike number. Both traits are highly correlated (0.88) with each other.

Seedling root weight (Figure 6g). There was strong, positive correlation between *TaCKX9* (*10*) expression in 7 DAP spikes and seedling root weight in non-silenced plants. Moreover, CKX activity negatively correlated with tZ (in spikes) and the trait, which finally resulted in lower root weight. The decreasing expression of *TaCKX11* (*3*) in the case of silent plants was positively correlated with decreasing content of cZOG and strongly positively correlated with the trait. Increasing expression levels of *TaCKX9* (*10*) plus *TaCKX2.2* negatively correlated with decreasing content of cZOG and root weight.

Chlorophyll content measured by SPAD in flag leaves of first spikes (Figure 6h). There was no correlation between expression level of any *TaCKX* measured in 7 DAP spikes of non-silent plants and the trait. The only correlations were between phytohormone content and the trait, positive for cZ and negative for GA, which resulted in higher SPAD values (chlorophyll content). Increasing expression of *TaCKX2.1* was strongly positively correlated with growing values of tZ, tZGs, cZ, and DZGs as well as GA in silent plants. A strong negative correlation was observed between the gene expression and chlorophyll content, which means that increasing expression of *TaCKX2.1* in 7 DAP spikes results in lower chlorophyll content in silent plants.

## 3. Discussion

First, 7 DAP spike was chosen as a research objective in wheat since decreased *HvCKX1* expression at this stage in barley resulted in higher yield due to the higher spike and grain number [20,21]. The *TaCKX1* gene is an orthologue of *HvCKX1* and both genes are specifically expressed in developing spikes [23], indicating their possibly important role in the regulation of yield-related traits. The samples were taken from the middle part of the spikes, when anthesis starts, in order to ensure a similar developmental stage of spikelets for research. The 7 DAP spikes of wheat represent the middle of cell division/cell expansion stage [35,36]. 

### 3.1. Various Levels of TaCKX1 Silencing Influence Different Models of Co-Expression with Other TaCKX Genes and Parameters of Yield-Related Traits 

Various levels of silencing of *TaCKX1* in T_1_ and T_2_ generate different results of co-expression with other *TaCKX* genes and plant phenotype. For example, the expression of *TaCKX9* (*10*) was highly and significantly correlated with *TaCKX1* only in T_1_. However, a new and strong positive correlation between *TaCKX9* (*10*) and *TaCKX2.2* in highly silenced T_2_ was observed. Slightly decreased co-expression of silenced *TaCKX1* together with *TaCKX11* (*3*) in T_1_ was much stronger in T_2_, indicating their positive co-regulation. It should be underlined that there is no homology between the sequence of *TaCKX1* used for silencing and sequences of other *TaCKX* genes tested. Therefore, the process of RNAi silencing was specifically addressed to *TaCKX1* silencing. It indicates that the level of silencing of the modified gene affected variable levels of expression of the other *TaCKX* genes in a co-operative process maintaining homeostasis of CKX enzyme in the research object. The models of co-regulation of other *CKX* by highly silenced *TaCKX1* and knock-out *HvCKX1* [27] differ between these species.

The differences in the levels of expression of *TaCKX1* and various co-expression of other *TaCKX* genes in T_1_ and T_2_ resulted in opposite phenotypic effects. Since spike number, grain number, and grain yield were reduced in T_1_, the same yield-related traits were significantly higher in highly silenced T_2_ plants. High-yielding phenotype occurred when highly silenced *TaCKX1* co-operated with down-regulated *TaCKX11* (*3*) but up-regulated *TaCKX5*, *TaCKX2.2*, *TaCKX2.1*, and *TaCKX9* (*10*). These differences showed that both levels of silencing might be helpful to better understand the function of developmentally regulated genes. Unexpectedly, changes in the expression levels of co-working *TaCKX* did not result in different enzyme activity, even in highly silenced T_2_ plants. This might be explained by the fact that down-regulation of *TaCKX1* and *TaCKX11* (*3*) is compensated for by the up-regulation of *TaCKX2.2*, *TaCKX5*, and *TaCKX9* (*10*), and therefore the contribution of isozymes encoded by the genes in the general pool of CKX enzyme activity is the same. Since CKX enzymes indicate different specificities for the particular cytokinin hormone [37], the cytokinin contribution and phenotypic traits of modified plants were changed accordingly, with consequent differences in the active pool of CKs influencing phenotype.

### 3.2. Co-Operating Effect of TaCKX on the Level of Active CKs in Silenced Plants

Since CKX isozymes specifically degrade CKs, the highly decreased expression of *TaCKX1* and *TaCKX11* (*3*) in 7 DAP spikes is expected to result in the observed increase of most major forms of CKs: tZGs, tZ, and cZ in silenced plants. We documented that both tZ and cZ, which are isomers of zeatin, together with their derivatives are a major group of isoprenoid CKs in 7 DAP spikes. It has already been shown that trans-zeatin is the predominant form after anthesis [36,38], but comprehensive analysis of cytokinins during spike, spikelet, ovule and grain development has not yet been reported for wheat using LC-MS/MS [22]. The content of DZGs increased by 40% in silent compared to non-silent wheat plants, suggesting that this less known isoprenoid form of CKs might also play an important role in plant productivity. Interestingly, isoprenoid iP was represented in 7 DAP spikes of non-silent plants at very low quantities, but its content in 7 DAP spikes of silent plants was increased by 32%. A similar relationship between the reduced expression of selected *CKX* family genes and cytokinin accumulation in reproductive organs has been observed in other species including *A*. *thaliana* [39], rice [19], and barley [25], but detailed data are not comparable to our research in wheat. 

The physiological significance of these isoprenoid forms is still not very well known. tZ and iP, which are susceptible to CKX, were found the most abundant and bioactive CKs in maize, whereas cZ, which shows low affinity to CKX was reported to have a weak biological impact and unknown biological role [40,41]. However, the cZ concentrations changed significantly during development in maize grain, as well as in shoot and root tissues [42,43]. High levels of cZ at the first developmental stage of barley spike observed by Powell et al. [44] might indicate an important role of this form in early barley embryo development, what is also documented in our results (discussed further below). 

The BA is represented in 7-DAP spikes of wheat at trace amounts but their content was significantly decreased in silent plants. However, their correlations with the *TaCKX* genes as well as yield-related traits of non-silenced plants indicate their importance (discussed in more detail below). Interestingly, BA was found to participate in posttranscriptional and/or posttranslational regulation of protein abundance in *Arabidopsis*, showing high specificity to shoots and roots, and affected differential regulation of hormonal homeostasis [45]. 

### 3.3. Cross Talk of CKs with Other Phytohormones

Negative correlations between ABA content and *TaCKX2.2* and *TaCKX9* (*10*) expression, and positive with *TaCKX11* (*3*), were associated with a slight decrease of ABA content in 7 DAP spikes of silenced plants. Moreover, ABA was strongly positively correlated with BA. The main auxin, IAA, remained at the same level. A ten-fold increase of GA content in silenced comparing to non-silenced plants was observed. Such cross regulation of CKs and other plant hormones is documented in other species. In maize kernels the *CKX1* gene is up-regulated by cytokinin and ABA, and abiotic stress [18]. In tobacco altered cytokinin metabolism affected cytokinin, auxin, and ABA contents in leaves and chloroplasts [46], which host the highest proportion of CK-regulated proteins [47]. Moreover, auxin, ABA and cytokinin are involved in the hormonal control of nitrogen acquisition and signalling [48], which often limits plant growth and development. All four phytohormones, CKs, GA, IAA, and ABA, were found to be involved in the regulation of grain development in drought conditions [49]. Moreover, in shoots, BA up-regulated the abundance of proteins involved in ABA biosynthesis and the ABA response, whereas in the roots, BA strongly up-regulated the majority of proteins in the ethylene biosynthetic pathway [45]. We proved that IAA, GA, and ABA contents are also co-regulated by CKs in non-silenced and silenced 7 DAP spikes. Up-regulation of major CKs and down-regulation of some minor ones in silent plants influence GA, ABA, and IAA content in a similar manner as in abiotic stress conditions.

### 3.4. Coordinated Effect of TaCKX Gene Expression on the Content of CKs, Other Phytohormones and Yield-Related Traits

Plant height in non-silenced plants is down-regulated by BA and up-regulated by IAA and GA content in the first 7 DAP spikes, resulting in taller plants. Oppositely, increased content of tZ and tZGs negatively correlated with the trait in silent plants, stimulated plant height. As it was already showed [50,51] and similarly to our results, plant height and root weight are regulated by CKs and IAA in opposite ways. This may be dependent on basipetal auxin flow in the stem, which suppresses axillary bud outgrowth, and similarly as in pea, auxin derived from a shoot apex suppresses the local level of CKs in the nodal stem through the regulation of *CKX* or *IPT* genes [52]. 

The main role in spike length seemed to be played by cZ and its glucoside. Increased content of cZOG in non-silenced plants negatively correlated with ABA, resulting in longer spikes. In silent plants the trait is positively regulated by *TaCKX2.2* together with *TaCKX5*, and the latter is a positive regulator of enzyme activity and negative of cZ content. Consequently, a higher content of cZ in 7 DAP spikes led to shorter spikes. cZOG, found as a positive regulator of longer spikes, is a sugar conjugate of cZ-0-glucoside, which is the inactivated form of cZ, showing metabolic stability against CKX activity [53]. Moreover, 0-glucosylation of cZ is catalysed by a specific 0-glucosyltransferase, cisZOG1, discovered in maize [54], and this form mainly functions in the early stages of seed development. Knowledge of function of cZ degradation pathways via the CKX enzyme is limited. Interestingly, two *Arabidopsis* genes, *CKX1* and *CKX7*, expressed in stages of active growth, were shown to have high preference for cZ [37]. In our case the *TaCKX5* positively regulated CKX activity and negatively cZ content.

None of the tested individual *TaCKX* genes was involved in high TGW in non-silenced plants, but a negative correlation with cZ and positive with GA was found. Otherwise a significant negative correlation of *TaCKX2.1* and a positive correlation of *TaCKX11* (*3*) in determining low TGW were observed in silenced plants. Unexpectedly increased expression of the first one positively influenced tZ, cZ, and iP content and negatively GA content, and the opposite was true for the second gene, resulting in lower TGW. Therefore both *TaCKX2.1* and *TaCKX11* (*3*), acting in an opposite manner, maintain homeostasis of CKX enzyme activity and co-regulate TGW in silenced plants. A greater concentration of CKs, especially tZ, was observed during the grain filling stage of high-yielding cultivars [44]. We might suppose that the observed higher concentrations of tZ and other CKs at the 7 DAP stage, which originally was a consequence of *TaCKX1* silencing, might accelerate germination of the grains, which resulted in smaller grains/lower TGW than in non-silenced plants. The silenced *TaCKX1* co-work with down-regulated *TaCKX11* (*3*) in increasing CK content as well as up-regulating *TaCKX2.1*, with seems to play a regulatory role. The involvement of GA in TGW and other traits demonstrated by us might be the effect of co-regulation of *CKX* and other gibberellin-responsive genes regulating yield-related traits as well [55,56]. Fahy et al. [57] suggested that final grain weight might be largely determined by developmental processes prior to grain filling. This is in agreement with our observations, in which yield-related traits are differently regulated in two groups of plants, non-silent and silent. Therefore, we might suppose that the coordinated co-regulation of expression of *TaCKX* genes and related CKs takes place during whole plant and spike development and small seeds in silenced plants are determined at earlier stages. 

Grain yield, which is very strongly correlated with grain and spike number in non-silent plants but with TGW in silent plants, is a more complex feature. Two groups of genes up-regulating or down-regulating grain yield in non-silent plants have been found. The first one includes *TaCKX1*, *2.2*, and *5* positively regulating iP content but negatively BA. The second comprises *TaCKX11* (*3*) acting in down-regulation of tZGs. Both groups might determine lower grain yield. It is worth to mention that *TaCKX5*, which is highly expressed in inflorescences and leaves might be a main player of this trait. Higher grain yield was positively regulated by enzyme activity and both, down-regulated *TaCKX11* (*3*) as well as up-regulated *TaCKX2.1* in silenced plants. Again, the *TaCKX2.1* positively regulated tZGs and cZ content just like for TGW, which is rather untypical for a gene encoding a CKX enzyme degrading CKs. Therefore, the positive regulation of the main CK content by *TaCKX2.1* observed by us supports its role in regulation of expression of other genes rather than encoding the CKX isozyme.

As observed in barley cultivars, changes in cytokinin form and concentration in developing kernels correspond with variation in yield [44]. Interestingly, the authors observed no peaks and no differences in CKX activity at the particular stages of spike development. This is in agreement with the homeostasis of the pool of isozymes in 7 DAP spikes of wheat, as suggested by us, which is independent of the level of silencing of *TaCKX1* but is rather a consequence of co-regulation of expression of other *TaCKX* genes. A similar effect of increased grain yield, which was a consequence of higher spike and grain number, was obtained in barley with silenced by RNAi *HvCKX1*, an orthologue of *TaCKX1* [20,21,25]. In this research, CKX activity was decreased, however according to Zalewski et al. [20], it was measured not in 7 DAP spikes, but in 0 DAP spikes and seedling roots. Therefore this inconsistency might be result of measurements in various organs/developmental stages. Another explanation is that these two cereal species varied three times in ploidy level, what might influence differences in action of both orthologues. The *TaCKX* homologues located on A, B and D chromosomes might significantly affect homeostasis of pooled CKX isozymes in wheat. Incomparable to the results obtained for RNAi silenced *TaCKX1* and *HvCKX1*, no changes in yield parameters were observed in mutant lines with knock-out of *HvCKX1* (Gasparis et al., 2019). These essential phenotypic differences between RNAi-silenced *TaCKX1* and *HvCKX1* or knocked out by CRISPR-Cas9 *HvCKX1* might be the result of different processes involved in inactivation of the gene. The first one is regulated at the posttranscriptional and the second at the transcriptional level. Since CKs might regulate various developmental and physiological processes at the posttranscriptional level [6,7] or by modulation of context-dependent chromatin accessibility [8], the way of deactivating *TaCKX* function seemed to be important.

Spike number and grain number are highly correlated in both non-silent and silent plants and are regulated by the same groups of *TaCKX* genes as well as phytohormones. The first group includes *TaCKX1*, *2.2* and *5* positively regulating iP but negatively BA. The second comprises *TaCKX11* (*3*) and *2.1* acting in the opposite way, and homeostasis of these hormones in non-silenced plants maintains a lower spike number. The main role in controlling higher spike and grain number in silent plants seemed to be played by *TaCKX5*, highly expressed in seedling roots, leaves, inflorescences and 0 DAP spikes. These correlations are not significant because they were measured in a stage of plant development in which the number of spikes and seed number have already been set. As reported, the higher spike number was the consequence of a higher tiller number, which was positively correlated with the content of endogenous zeatin in the field-grown wheat after exogenous hormonal application [58]. Shoot branching might also be dependent on the acropetal transport of cytokinin [52]. 

Root weight was positively correlated with lower expression of *TaCKX9* (*10*) in 7 DAP spikes of non-silent plants and, negatively with increased expression of this gene in silenced plants. Therefore the gene might determine lower root weight in the first group of plants, but higher in the second. Increased expression of *TaCKX9* (*10*) down-regulated cZOG. The same cZOG was up-regulated by *TaCKX11* (*3*), but expression of this gene in 7 DAP spikes of silent plants is strongly decreased. Both cZ and cZOG are involved in spike length regulation as well as TGW and grain yield in the group of silenced plants. Although both tested organs are in different developmental stages, correlations between *TaCKX9* (*10*) and *TaCKX11* (*3*) expression in 7 DAP spikes and weight of seedling roots are reasonable. The *TaCKX9* (*10*) is mainly expressed in younger organs from seedling roots to 0 DAP spikes and highly expressed in leaves. The *TaCKX11* (*3*) is expressed in all organs tested [23] and both seemed to regulate seedling roots as well, although in the opposite manner. Therefore, we should take into consideration the possible action of cytokinin transport and signalling genes as well as other phytohormones which take part in hormonal crosstalk to control the regulation of root growth [59]. Accordingly, cZ type CKs found as the major forms in phloem are translocated from shoots to roots [60,61]. Some *CKX* genes might be induced by transcription factors [62,63], what is also observed in our unpublished yet data.

The lower plant height and higher root weight observed in the group of silenced plants of wheat is in agreement with opposed regulation of these traits by CKs and IAA mentioned above [64,65]. Up-regulated content of active cZ in 7 DAP spikes, might influence down-regulation of this CK in roots. It has been documented that such suppressing cZ levels mediated by overexpression of *AtCKX7* affected root development in *Arabidopsis* [66]. A higher weight of seedling root was also obtained by silencing via RNAi or knock-out via CRISPR/Cas9 of *HvCKX1* in barley plants, as in wheat, and the trait corresponded with decreased activity of CKX enzyme measured in roots (Zalewski et al., 2010; Gasparis et al., 2019).

Leaf senescence was determined in the flag leaf of the first spike by measuring chlorophyll content. Increased expression of *TaCKX2.1* in silent plants up-regulated tZ, tZGs and cZ content in 7 DAP spikes and down-regulated the trait. The gene functions in a similar way, by up-regulating these CKs in determining lower TGW and higher grain yield in silent plants. A higher content of active CKs as well as GA in 7 DAP spikes of silent plants is expected to down-regulate CKs in the flag leaves, accelerating their senescence, what is documented by the results. 

It was previously demonstrated that level of chlorophyll content in flag leaves is associated with the senescence process, in which CKs suppress inhibition of senescence [67]. During this processes, proteins are degraded and nutrients are re-mobilised from senescing leaves especially to the developing grains [68]. We might suppose that slower spike ripening in non-silent plants, which is dependent on lower CK content in the 7 DAP spike, causes a slower flow of micronutrients as well as CKs from flag leaf to spike. Therefore, prolonged chlorophyll content in the flag leaf of the first spike negatively correlated with TGW but positively with plant height. Opposite data were obtained for flag leaves of silent plants, in which higher content of CKs in 7 DAP spikes might be the result of faster flow accelerating leaf senescence. The reduced chlorophyll content in flag leaves of the first spike of silent plants positively correlated with grain yield. The important role of tZ and less active cZ in the suppression of senescence was proven in maize leaves [69] and in an oat-leaf assay [37]. It was also documented that delayed senescence of wheat stay-green mutant, tasg1, at the late filling stage was related to high cytokinin and nitrogen contents [70]. 

## 4. Materials and Methods 

### 4.1. Vector Construction

The hpRNA type of silencing cassette was constructed in pBract207 (https://www.jic.ac.uk/technologies/crop-transformation-bract/). It contains the Hpt selection gene under the 35S promoter and cloning sites for the cloning silencing cassette under the Ubi promoter. The vector is compatible with the gateway cloning system. For cloning purposes a coding sequence of *TaCKX1* (NCBI JN128583) 378 codons long was used. In the first step, the cassette was amplified using: EAC11-F: 5′-TTGAATTCGACTTCGACCGCGGCGTTTT-3′ and EAC12-R: 5′-TTGAATTCATGTCTTGGCCAGGGGAGAG-3′ and cloned into the entry vector pCR8/GW/TOPO (Invitrogen). In the next step, the cassette was cloned to the destination Bract7 vector in the gateway reaction. The presence of the silencing cassette in the vector was verified by restriction analysis and sequencing. The vector was electroporated into the AGL1 strain of *Agrobacterium tumefaciens* and used for transformation.

### 4.2. Plant Material, Agrobacterium-Mediated Transformation and In-Vitro Culture

The spring cultivar of common wheat (*Triticum aestivum* L.) Kontesa was used as a donor plant for transformation experiments as well as transgenic plants. Seeds were germinated into Petri dishes for one day at 4 °C and then five days at room temperature in the dark. Six out of ten seedlings from each Petri dish were replanted into pots with soil. The plants were grown in a growth chamber under controlled environmental conditions with 20 °C/18 °C day/night temperatures and a 16 h light/8 h dark photoperiod. The light intensity was 350 µmol·s^−1^·m^−2^.

*Agrobacterium*-mediated transformation experiments were performed according to our previously described protocols for wheat [71,72]. Putative transgenic plants were regenerated and selected on modified MS media containing 25 mg·L^−1^ of hygromycin as a selectable agent.

First, 7 days after pollination, (DAP) spikes from T_1_, T_2_, and control plants were collected for RT-qPCR and phytohormone quantification. Only 1 in 3 of the middle part of each spike was used for experiments (upper and lower parts were removed).

### 4.3. PCR Analysis

Genomic DNA was isolated from well-developed leaves of 14-day plants according to the modified CTAB procedure [73] or by using the KAPA3G Plant PCR Kit (Roche Sequencing and Life Science, Kapa Biosystems, Wilmington, MA, USA). The PCR for genomic DNA isolated by CTAB was carried out in a 25 mL reaction mixture using Platinum Taq DNA Polymerase (Invitrogen by Thermo Fisher Scientific, Waltham, MA, USA) and 120 ng of template DNA. The reaction was run using the following program: initial denaturation step at 94 °C for 2 min, 35 cycles of amplification at 94 °C for 30 s, 65 °C for 30 s, 72 °C for 30 s with a final extension step at 72 °C for 5 min. The PCR for genomic DNA isolated by KAPA3G was carried out in a 50 μL reaction mixture using 1 U of KAPA3G Plant DNA Polymerase and a 0.5 × 0.5 mm leaf fragment. The reaction was run using the following program: initial denaturation step at 95 °C for 3 min, 40 cycles of amplification at 95 °C for 20 s, 68 °C for 30 s, 72 °C for 30s with a final extension step at 72 °C for 2 min.

Putative transgenic T_0_ and T_1_ plants were tested with two pairs of specific primers amplifying a fragment of the *hpt* selection gene. The sequences of the primers for the first pair were: hygF1 5′-ATGACGCACAATCCCACTATCCT-3′ and hygR1 5′-AGTTCGGTTTCAGGCAGGTCTT-3′, and the amplified fragment was 405 bp. The sequences of the primers for the second pair were: hygF2 5′-GACGGCAATTTCGATGATG-3′ and hygR2 5′-CCGGTCGGCATCTACTCTAT-3′, and the amplified fragment was 205 bp. 

Non-transgenic null segregants were used as a control.

### 4.4. RNA Extraction and cDNA Synthesis

Total RNA from 7 DAP spikes was extracted using TRI Reagent (Sigma-Aldrich, Hamburg, Germany) and 1-bromo-3-chloropropane (BCP) (AppliChem GmbH, Darmstadt, Germany) according to the manufacturer’s protocol. The purity and concentration of the isolated RNA were determined using a NanoDrop spectrophotometer (NanoDrop ND-1000) and the integrity was checked by electrophoresis on 1.5% (w/v) agarose gels. To remove the residual DNA the RNA samples were treated with DNase I, RNase-free (Thermo Fisher Scientific, Waltham, MA, USA). Each time 1 µg of good quality RNA was used for cDNA synthesis using the RevertAid First Strand cDNA Synthesis Kit (Thermo Fisher Scientific) following the manufacturer’s instructions. The obtained cDNA was diluted 20 times before use in RT-qPCR assays.

### 4.5. Quantitative RT-qPCR 

RT-qPCR assays were performed for 6 target genes: *TaCKX1* (JN128583), *TaCKX2.1* (JF293079)/*2.2* (FJ648070), *TaCKX11* (*3*) (JN128585), *TaCKX5* (Lei et al., 2008), *TaCKX9* (*10*) (JN128591). Primer sequences designed for each gene as well as for the reference gene are shown in Table S1. All real-time reactions were performed in a Rotor-Gene Q (Qiagen) thermal cycler using 1× HOT FIREPol EvaGreen qPCR Mix Plus (Solis BioDyne), 0.2 µM of each primer, and 4 µL of 20 times diluted cDNA in a total volume of 10 µL. Each reaction was carried out in 3 technical replicates at the following temperature profile: 95 °C—15 min initial denaturation and polymerase activation (95 °C—25 s, 62 °C—25 s, 72 °C—25 s) × 45 cycles, 72 °C—5 min, with the melting curve at 72–99 °C, 5 s per step. The expression of *TaCKX* genes was calculated according to the two standard curves method using ADP-ribosylation factor (*Ref 2*) as a normalizer.

Relative expression/silencing of *TaCKX1* was related to mean expression of the gene in non-silenced control plants set as 1.00. Relative expression of other *TaCKX* genes was related to each tested gene set as 1.00 in non-silenced plants.

Statistical analysis was performed using Statistica v13.3 software (StatSoft, Kraków, Poland). The normality of data distribution was tested using the Shapiro–Wilk test. To determine whether the means of two sets of data of expression levels, phytohormone concentrations, and yield-related traits between non-silenced and silenced lines are significantly different from each other (for *p* value less than *p* < 0.05), either the Student’s *t*-test or the Mann–Whitney test was applied. Correlation coefficients were determined using parametric correlation matrices (Pearson’s test) or a nonparametric correlation (Spearman’s test).

### 4.6. Quantification of ABA, Auxins, Cytokinins and GA_3_

Chemicals used for quantification were: the standard of ABA, five standards of auxins: IAA, indole-3-butyric acid (IBA), indole-3-propionic acid (IPA), 1-naphthaleneacetic acid (NAA), and 2-phenylacetic acid (PAA); twenty-seven standards of CKs: tZ, *trans*-zeatin riboside (tZR), *trans*-zeatin-9-glucoside (tZ9G), *trans*-zeatin-7-glucoside (tZ7G), *trans*-zeatin-*O*-glucoside (tZOG), *trans*-zeatin riboside-*O*-glucoside (tZROG), *trans*-zeatin-*9*-glucoside-*O*-glucoside (tZ9GOG), *trans*-zeatin-9-glucoside riboside (tZ9GR), *c*Z, *cis*-zeatin-riboside (cZR), *cis*-zeatin *O*-glucoside (cZOG), *cis*-zeatin 9-glucoside (cZ9G), *cis*-zeatin-*O*-glucoside-riboside (cZROG), dihydrozeatin (DZ), dihydrozeatin-riboside (DZR), dihydrozeatin-9-glucoside (DZ9G), dihydrozeatin-7-glucoside (DZ7G), dihydrozeatin-*O*-glucoside (DZOG), dihydrozeatin riboside-*O*-glucoside (DZROG), *N*^6^-isopentenyladenine (iP), *N*^6^-isopentenyladenosine (iPR), *N*^6^-isopentenyladenosine-7-glucoside (iP7G), *para*-topolin (*p*T), *meta*-topolin (*m*T), *ortho*-topolin (*o*T), 6-benzylaminopurine (6-BAP), and standard of GA_3_. 

For the measurement of phytohormones, 200 mg of plant powders were placed into the 2-mL Eppendorf tubes, suspended in 1 mL of (*v*/*v*) 50% ACN, and homogenized in a bead mill (50 Hz, 5 min) using two 5-mm tungsten balls. Then, samples were homogenized using the ultrasound processor VCX 130 (max. power 130 W, max. frequency 20 kHz, 5 min) equipped with titanium probe and mixed in laboratory shaker (90 rpm, dark, 5 °C, 30 min). Samples were centrifuged (9000× *g*, 5 min) and collected in a glass tube. For the quantification of ABA, AXs, CKs, and GA_3_, [^2^H_6_](+)-*cis*,*trans*-ABA (50 ng), [^2^H_5_] IAA (15 ng), [^2^H_6_] iP (50 ng), [^2^H_5_] *t*Z (30 ng), [^2^H_5_]-*t*ZOG (30 ng), [^2^H_3_]-DZR (30 ng), and [^2^H_2_] GA_3_ (30 ng) were added to samples as internal standards.

Prepared extracts were purged using a Waters SPE Oasis HLB cartridge (Waters Corporation, Milford, MA, USA), previously activated and equilibrated using 1 mL of 100% MeOH, 1 mL water, and 1 mL of (*v*/*v*) 50% ACN [74]. Then, extracts were loaded and collected to the Eppendorf tubes and eluted with 1 mL of 30% ACN (*v*/*v*). Samples were evaporated to dryness by centrifugal vacuum concentrator, dissolved in 50 µL of (*v*/*v*) 30% ACN and transferred into the insert vials. Detection of analyzed phytohormones was performed using an Agilent 1260 Infinity series HPLC system (Agilent Technologies, Santa Clara, CA, USA) including a Q-ToF LC/MS mass spectrometer with Dual AJS ESI source; 10 μL of each sample was injected on the Waters XSelect C_18_ column (250 mm × 3.0 mm, 5 μm), heated up to 50 °C. Mobile phase A was 0.01% (*v*/*v*) FA in ACN and phase B 0.01% (*v*/*v*) FA in water; flow was 0.5 mL min^−1^. Separation of above hormones was done in ESI-positive mode with the following gradient: 0–8 min flowing increased linearly from 5 to 30% A, 8–25 min 80% A, 25–28 min 100% A, 28–30 min 5% A.

For the optimization of MS/MS conditions, the chemical standards of analyzed phytohormones were directly injected to the MS in positive ([M + H]^+^) ion scan modes, then areas of detected standard peaks were calculated. [M + H]^+^ was chosen because of its significantly better signal-to-noise ratios compared to the negative ion scan modes.

Chlorophyll content was measured using an SPAD chlorophyll meter.

## 5. Conclusions

Based on the 7 DAP spike as a research object, we have documented that silencing of *TaCKX1* by RNAi strongly influenced up- or down-regulation of other *TaCKX* genes, as well as phytohormone levels and consequently phenotype. This co-regulation is dependent on the level of silencing of the gene and is independent of cross-silencing of other *TaCKX* genes. Detailed analysis revealed that each tested yield-related trait is regulated by various up- or down-regulated *TaCKX* genes and phytohormones. Key genes involved in the regulation of grain yield, TGW, or root weight in highly silenced plants are *TaCKX2.1* and *TaCKX11* (*3*) acting antagonistically, and increased expression of the first one determines growth of tZ, tZ derivatives, and cZ, whereas decreased expression of the second down-regulates content of cZOG. A key role in determination of the high-yielding phenotype seemed to be played by the growing content of tZ in 7 DAP spikes, which might accelerate maturation of immature grains by speeding up nutrient flow from flag leaves. This finally led to reduction of TGW but enhancement of grain number and yield. The latter traits are the result of a higher spike number, which is determined in the early stages of plant development.

## Figures and Tables

**Table 1:** Effect of *TaCKX1* silencing on expression levels of selected *TaCKX* genes presented by expression ratio indicator (mean value in silent/mean value in non-silent, control plants) in T_1_ and T_2_ generations.

	T_1_ (SD)	T_2_ (SD)	Effect of *TaCKX1* Silencing T_1_/T_2_
*TaCKX1* *	0.58 (0.12)	0.28 (0.05)	decreased/strongly decreased
*TaCKX11 (3)*	0.80 (0.16)	0.36 (0.05)	decreased/strongly decreased
*TaCKX2.2*	1.08 (0.22)	0.98 (0.18)	slightly increased/similar
*TaCKX9 (10)*	0.59 (0.20)	1.15 (0.32)	strongly decreased/slightly increased
*TaCKX2.1*	1.22 (0.19)	1.32 (0.35)	increased/increased
*TaCKX5*	1.00 (0.65)	1.08 (0.52)	the same/similar
CKX activity	1.01 (0.07)	0.99 (0.18)	the same/the same

*—significant at *p* < 0.05.

## Supplementary Materials

Supplementary materials can be found at https://www.mdpi.com/1422-0067/21/13/4809/s1. Table S1: Primer sequences designed for reference gene and each of 6 tested *TaCKX* genes and amplicon length. Table S2: Phenotypic traits and ratio indicator in silent T_1_ and not silent, control plants. Table S3: Phenotypic traits and ratio indicator in silent T_2_ and not silent, control plants. Table S4: A. B. Correlation coefficients among expression of all tested *TaCKX* genes and enzyme activity, and phenotypic traits in not-silent (A) and highly silent T_2_ plants (B). * non-parametric analysis; in bold: significant at *p* < 0.01.
