# Supplementary material for: Silencing of TaCKX1 Mediates Expression of Other TaCKX Genes to Increase Yield Parameters in Wheat
Source: Int J Mol Sci. 2020 Jul 7;21(13):4809. doi: 10.3390/ijms21134809 (PMC7369774; doi:10.3390/ijms21134809)
Supplement: Supplementary file 1 [file ijms-21-04809-s001.pdf]

Table S1. Primer sequences designed for reference gene and each of 6 tested *TaCKX* genes and amplicon length.

Gene	Forward primer	Reverse primer	Amplicon length
Ref2	GCTCTCCAACAACATTGCCAAC	GCTTCTGCCTGTCACATACGC	165
TaCKX1	GTCTACCCGCTCAACAAATCC	CCCAGGTACTCCTTGTACCCTAT	188
TaCKX2.1	TCTACCCCATGAACCGGGAC	TCTCCTCGTTCTGCTCCTCC	144
TaCKX2.2	TTGATCGCGGAGCTAATCCA	TATCACATACGCCATCCATGC	205
TaCKX3	TTGTCAAGGGACTGTAGTAGGG	GAATTAGAGTTCACGGCTTGATG	150
TaCKX5	GTCCGATTTTTGAGAAGACTGATT	CATACATGACACCAACGTACATCTT	150
TaCKX10	GAGCTAAGGGCTTGTGGGA	ACATAAAGCAATTTACCTGGACTTG	167

Table S2. Phenotypic traits and ratio indicator in silent T_1 and non-silent, control plants.

T_1 (mean expression of *TaCKX1*)	plant height (cm) [SD]	number of spikes [SD]	spike length [SD]	grain number [SD]	grain yield (g) [SD]	TGW (g) [SD]	SPAD first spike [SD]	SPAD next spikes [SD]
Silent (0.58)	71.00 [3.91]	3.88 [1.27]	9.58 [0.98]	125.00 [47.88]	5.64 [1.93]	45.87 [3.2]	53.75 [1.55]	46.48 [5.67]
Non-silent (1.00)	71.00 [2.69]	4.88 [1.05]	9.94 [0.59]	178.13 [48.48]	7.91 [2.39]	44.36 [4.92]	53.39 [3.37]	46.81 [7.26]
Ratio indicator[1]	1.00	0.79	0.96	0.70	0.71	1.03	1.05	0.99

[1] ratio indicator (mean value in silent / mean value in not silent, control plants)

Table S3. Phenotypic traits and ratio indicator in silent T_2 and non-silent, control plants.

T_2 (mean expression of *TaCKX1*)	plant height (cm) [SD]	number of spikes [SD]	spike length [SD]*	grain number [SD]	grain yield (g) [SD]	TGW (g) [SD]*	SPAD first spike [SD]	SPAD next spikes [SD]	Root weight (mg) [SD]
Silent (0.28)	70.33 [5.06]	7.83 [2.54]	7.78* [0.96]	127.33 [44.69]	4.80 [2.01]	36.59* [9.24]	40.10 [3.65]	44.08 [16.8]	92.00 [15.89]
Non-silent (1.00)	76.00 [3.51]	5.00 [1.91]	9.06* [0.68]	98.50 [40.43]	4.65 [1.93]	47.09* [2.01]	42.27 [3.13]	40.33 [4.56]	86.00 [18.00]
Ratio indicator	0.93	1.57	0.86	1.29	1.03	0.78	0.95	1.09	1.07

* significantly different ($p \leq 0.05$)

[1] ratio indicator (mean value in silent / mean value in non-silent, control plants)

Table S4 A. B. Correlation coefficients among expression of all tested *TaCKX* genes and enzyme activity, and phenotypic traits in non-silent (A) and highly silent T_2 plants (B). * non-parametric analysis; in bold - significant at $p<0.01$

silent	spike number*	spike length	grain number*	grain yield (g)	TGW	root mass	CKX activity	*TaCKX 1*	*TaCKX 2.1*	*TaCKX 2.2*	*TaCKX 11 (3)*	*TaCKX 5**	*TaCKX 9 (10)*	tZ	tZGs	cZ	cZOG	DZGs	iP	BA	IAA	ABA	GA	SPAD first spike
**plant height**	0.03	0.31	0.38	0.34	0.32	-0.26	0.11	-0.32	-0.52	0.25	0.06	-0.15	0.43	-0.64	-0.7	-0.19	-0.24	-0.19	-0.45	0.3	-0.47	0.31	-0.22	0.42
**spike number***		-0.03	**0.88**	-0.33	-0.39	-0.33	-0.03	-0.52	0.52	-0.21	-0.33	0.52	-0.15	0.52	-0.15	0.39	0.03	0.52	0.76	0.58	0.46	0.15	0.58	-0.27
**spike length**			0.2	0.61	0.55	0.12	**0.85**	0.33	-0.38	0.62	0.14	0.77	0.41	-0.42	**-0.82**	-0.62	-0.04	-0.52	-0.37	-0.48	0.13	-0.43	-0.53	0.52
**grain number***				0.03	-0.09	-0.2	0.2	-0.54	0.14	-0.26	-0.14	0.6	-0.14	0.09	-0.54	0.03	0.26	0.26	0.43	0.54	0.54	0.14	0.2	0.2
**grain yield (g)**					**0.82**	0.66	0.81	-0.16	-0.6	-0.21	0.75	0.03	-0.34	-0.42	-0.76	-0.66	0.6	0.03	-0.53	0.07	0.36	0.39	-0.59	0.61
**TGW**						0.77	0.53	0.24	**-0.85**	-0.2	0.79	0.2	-0.33	-0.61	-0.55	-0.78	0.59	-0.27	**-0.9**	-0.33	0.09	0.22	-0.77	0.58
**root mass**							0.36	0.13	-0.54	-0.65	**0.85**	0.26	**-0.82**	-0.2	-0.06	-0.59	**0.91**	0.08	-0.57	-0.21	0.46	0.35	-0.58	0.34
**CKX activity**								0.03	-0.24	0.25	0.37	0.77	0.01	-0.19	-0.78	-0.56	0.28	-0.12	-0.16	-0.15	0.47	-0.1	-0.44	0.49
TaCKX1									0.02	0.39	0.12	0.2	0.14	0.14	0.19	-0.06	-0.23	-0.34	-0.29	-0.8	-0.35	-0.59	-0.04	-0.32
TaCKX2.1										0.18	-0.47	0.14	0.13	**0.91**	0.52	**0.84**	-0.52	0.52	**0.93**	0.29	-0.06	-0.18	**0.89**	-0.8
TaCKX2.2											-0.58	0.03	**0.93**	-0.09	-0.38	0.02	-0.76	-0.54	0.14	-0.42	-0.31	-0.76	0.07	0.00
TaCKX11 (3)												0.26	-0.73	-0.09	-0.22	-0.34	0.66	0.35	-0.59	0.08	0.07	0.6	-0.32	0.12
TaCKX5*													-0.14	0.03	-0.26	-0.43	0.54	-0.26	0.37	-0.09	0.71	-0.43	-0.14	0.09
TaCKX9 (10)														-0.22	-0.34	0.09	**-0.82**	-0.54	0.16	-0.23	-0.41	-0.63	0.1	0.06
**tZ**															0.59	0.8	-0.28	0.71	0.76	0.28	-0.05	0.03	**0.85**	**-0.88**
**tZGs**																0.56	-0.04	0.3	0.39	-0.02	-0.08	-0.04	0.5	-0.67
**cZ**																	-0.61	0.66	0.71	0.52	-0.48	0.16	**0.99**	**-0.9**
**cZOG**																		0.07	-0.41	-0.02	0.69	0.42	-0.62	0.5
**DZGs**																			0.46	0.78	-0.06	0.71	0.68	-0.61
**iP**																				0.41	0.18	-0.14	0.75	-0.55
**BA**																					-0.06	0.79	0.51	-0.21
**IAA**																						-0.05	-0.43	0.48
**ABA**																							0.13	-0.02
**GA**																								**-0.91**

non-silent	spike number	spike length *	grain number	grain yield (g)	TGW	root mass	CKX activity *	*TaCKX 1**	*TaCKX 2.1**	*TaCK X2.2**	*TaCKX 11 (3)**	*TaCKX 5**	*TaCKX 9 (10)**	tZ	tZGs	cZ	cZOG	DZGs	iP	BA	IAA*	ABA	GA*	SPAD first spike
**plant height**	0.42	-0.29	0.43	0.49	0.7	-0.35	0.26	0.05	-0.15	0.05	-0.05	0.05	-0.26	0.09	-0.07	-0.08	-0.15	-0.05	0.55	-0.66	0.52	0.25	0.66	0.01
**spike number**		-0.53	**0.91**	**0.93**	0.33	0.29	-0.34	0.69	-0.55	0.69	-0.69	0.69	0.34	**0.84**	-0.05	0.17	-0.49	-0.1	**0.89**	**-0.95**	-0.09	0.3	0.4	-0.09
**spike length***			-0.54	-0.29	0.41	-0.03	-0.51	-0.31	0.51	-0.31	0.31	-0.31	0.51	-0.46	-0.06	-0.55	0.7	-0.06	-0.29	0.54	-0.29	-0.75	-0.27	-0.38
**grain number**				**0.99**	0.17	0.14	-0.26	0.77	-0.67	0.77	-0.77	0.77	0.26	0.71	0.15	0.36	-0.28	0.18	**0.95**	**-0.9**	-0.12	0.01	0.27	0.29
**grain yield (g)**					0.27	0.13	-0.34	0.68	-0.54	0.68	-0.68	0.68	0.34	0.7	0.1	0.28	-0.3	0.13	**0.97**	**-0.93**	-0.09	0.04	0.39	0.22
**TGW**						-0.15	-0.34	-0.14	0.27	-0.14	0.14	-0.14	0.34	-0.05	-0.36	-0.73	-0.12	-0.37	0.44	-0.43	0.2	0.14	0.65	-0.58
**root mass**							-0.68	-0.07	0.34	-0.07	0.07	-0.07	0.68	0.66	-0.75	-0.06	0.29	-0.74	0.11	-0.03	0.14	0.51	0.13	-0.14
**CKX activity***								-0.2	-0.2	-0.2	0.2	-0.2	-1.00	-0.51	0.34	0.34	-0.51	0.17	-0.34	0.26	0.34	0.34	0.00	0.51
TaCKX1*									**-0.92**	1.00	-1.00	1.00	0.2	0.51	0.54	0.54	-0.3	0.78	0.68	-0.57	-0.68	-0.27	-0.31	0.3
TaCKX2.1*										**-0.92**	**0.92**	**-0.92**	0.2	-0.3	-0.68	-0.68	0.51	**-0.85**	-0.54	0.46	0.54	0.14	0.31	-0.51
TaCKX2.2*											-1.00	1.00	0.2	0.51	0.54	0.54	-0.3	0.78	0.68	-0.57	-0.68	-0.27	-0.31	0.3
TaCKX11 (3)*												-1.00	-0.2	-0.51	-0.54	-0.54	0.3	-0.78	-0.68	0.57	0.68	0.27	0.31	-0.3
TaCKX5*													0.2	0.51	0.54	0.54	-0.3	0.78	0.68	-0.57	-0.68	-0.27	-0.31	0.3
TaCKX9 (10)*														0.51	-0.34	-0.34	0.51	-0.17	0.34	-0.26	-0.34	-0.34	0.00	-0.51
**tZ**															-0.27	0.36	-0.34	-0.33	0.59	-0.7	-0.03	0.59	0.39	-0.02
**tZGs**																0.53	-0.48	**0.97**	0.01	-0.08	-0.77	-0.58	-0.65	0.39
**cZ**																	-0.22	0.52	0.06	-0.22	-0.37	0.06	-0.65	0.73
**cZOG**																		-0.27	-0.19	0.52	0.03	-0.24	-0.39	0.4
**DZGs**																			0.06	-0.03	-0.71	-0.71	-0.65	0.55
**iP**																				**-0.89**	-0.09	-0.04	0.39	0.13
**BA**																					-0.12	-0.28	-0.53	0.01
**IAA***																						0.66	0.65	-0.09
**ABA**																							0.65	-0.36
**GA***																								-0.65
